# Resveratrol Improves Intestinal Morphology and Anti-Oxidation Ability in Deoxynivalenol-Challenged Piglets

**DOI:** 10.3390/ani12030311

**Published:** 2022-01-27

**Authors:** Qihua Hong, Xin Li, Qian Lin, Zhuojun Shen, Jie Feng, Caihong Hu

**Affiliations:** 1 College of Animal Sciences, Zhejiang University, Hangzhou 310058, China; lixin_andy@163.com (X.L.); linqian0824@163.com (Q.L.); shenzhuojun73@163.com (Z.S.); fengj@zju.edu.cn (J.F.); 2 Key Laboratory of Animal Nutrition and Feed Science (Eastern of China), Ministry of Agriculture and Rural Affairs, Hangzhou 310058, China; 3 Key Laboratory of Animal Feed and Nutrition of Zhejiang Province, Hangzhou 310058, China; 4 Key Laboratory of Molecular Animal Nutrition, Ministry of Education, Zhejiang University, Hangzhou 310058, China

**Keywords:** resveratrol, weaned piglets, deoxynivalenol (DON), growth performance, intestine, antioxidation

## Abstract

**Simple Summary:**

Deoxynivalenol (DON)-contaminated feed may cause anorexia, vomiting, immunosuppression, and intestinal dysfunction in pigs, which would lead to growth retardation and great losses in the pig industry. In this study, the effects of resveratrol (RES) on growth performance, the intestinal barrier, antioxidant capacity, and mitochondrial function in weaned pigs fed with DON-contaminated diets were investigated. Dietary supplementation with resveratrol increased the average daily feed intake of piglets. Diets supplemented with resveratrol increased the villus height and the ratio of the jejunum villus height to crypt depth, increased the activities of superoxide dismutase (SOD), and increased the total antioxidant capacity in the jejunum mucosa. After being supplemented with RES, the level of reactive oxygen species (ROS) in mitochondria was decreased, while the mitochondrial membrane potential in the jejunum was increased. In conclusion, these results suggested that resveratrol effectively relieved DON-induced oxidative stress in weaned piglets, improved intestinal barrier function, enhanced mitochondrial function, and improved the growth performance of piglets.

**Abstract:**

This study aimed to investigate the potential effects of resveratrol (RES) on intestinal function and oxidative stress in deoxynivalenol (DON)-challenged piglets. Twenty-four healthy Duroc × Yorkshire × Landrace weaned piglets at the age of 28 ± 1 days were randomly divided into four groups with six repetitions per group. The four groups were as follows: the control group (CON), fed with a basic diet; the RES group, fed with a basal diet + 300 mg/kg RES; the DON group, fed with a basal diet containing 2.65 mg/kg DON; and the DON + RES group, fed with a basal diet containing 2.65 mg/kg DON + 300 mg/kg RES. The results showed that the growth performance and intestinal function of DON-challenged piglets were significantly decreased (*p* < 0.05). Compared with the DON group, the average daily feed intake of piglets in the DON + RES group was significantly increased (*p* < 0.05). Additionally, dietary RES ameliorated DON-induced intestinal morphology impairment, as indicated by the increased (*p* < 0.05) jejunal villi height and the ratio of the jejunal villi height/crypt depth. Furthermore, after the addition of RES, the activities of superoxide dismutase (SOD) and total antioxidant capacity (T-AOC) in the jejunum mucosa were significantly increased, and the content of malondialdehyde (MDA) was significantly declined (*p* < 0.05). In addition, the level of reactive oxygen species (ROS) in the mitochondria was significantly reduced by RES, while the mitochondrial membrane potential in jejunum was significantly increased by RES (*p* < 0.05). However, there was no obvious difference between DON + RES and DON groups on average daily gain and the ratio of feed togain, except for the significant inhibition of average daily feed intake (*p* < 0.05). In conclusion, RES could effectively alleviate the DON-induced oxidative stress on weaned piglets, and reduce the damage to mitochondria and intestinal morphology, so as to improve the growth performance of piglets.

## 1. Introduction

Deoxynivalenol (DON), with a stable structure and bioactivity, is a toxic metabolite produced by *Fusarium graminearum*. It is widely found in cereal crops, such as wheat and corn, as well as their by-products [1]. Piglets are often at high risk of exposure to DON, because corn and wheat are common ingredients in their formulated feed. The main toxic effects of DON involve anorexia, vomiting, decreased growth performance, immunosuppression, intestinal dysfunction, and increased susceptibility to intestinal infectious diseases, resulting in large economic losses to the animal industry [2,3].

Physical, chemical, and biological methods are commonly used for the detoxification of DON. Presently, the addition of physical adsorbents into feed is the most popular method for detoxification. However, most of the adsorbents have low adsorption capabilities for DON due to their weak polarities and poor electrophilicities [4,5]. Because oxidative stress is regarded as an important mechanism of DON toxicity [6], extensive attention has been focused on substances that effectively inhibit oxidative stress and improve immune function [7,8]. Resveratrol (RES) is a naturally occurring polyphenol found in peanuts, red wine, grapes, pistachios, mulberries, and chocolate [9,10,11]. It has been shown that RES possesses efficient antioxidant activities both in vitro [12] and in vivo [13]. Supplementation with RES could significantly reverse the decline in the growth performance of piglets, and improve the feed efficiency of intrauterine growth-retarded suckling piglets [14].

Intestinal functions, such as the morphology of the villus and crypt, play a vital role in the growth performance of piglets [15]. Meanwhile, as a valid indicator of intestinal permeability in mammals, diamine oxidase (DAO) shows a high level of content and activity in the small intestinal villi, but low levels of content and activity in other tissues in normal conditions. However, when the intestinal mucosa is damaged, DAO will pass through into the blood, causing an increase in DAO activity in the plasma [16]. Lactic acid is a metabolic product of bacterial fermentation, which is rarely absorbed under normal circumstances, and cannot be degraded rapidly in mammals. As intestinal permeability increases, the D-lactic acid produced by intestinal bacteria will also enter the bloodstream through the damaged mucosa [17]. Hence, the levels of DAO and D-lactic acid in plasma usually reflect the degree of damage to the intestinal barrier, also called intestinal permeability. Moreover, it has been suggested that the addition of RES could protect the intestinal mucosal–epithelial barrier in an ischemia/reperfusion rat model because of its antioxidant capacity [13,18]. Nevertheless, information regarding the effect of RES on intestinal injuries induced by DON in piglets is unavailable. In the present study, 300 mg/kg RES was added to a diet containing 2.65 mg/kg DON to determine the role of RES on growth performance, intestinal barrier, and anti-oxidation in piglets challenged by DON.

## 2. Materials and Methods

### 2.1. Laboratory Animals and Diets

The Institutional Animal Care and Use Committee of Zhejiang University (Hangzhou, China) has approved all procedures for the experiment, with the permit number for conducting animal experiments of ZJU2018-118-44. Twenty-four weaned piglets (28 ± 1-day-old, Duroc × Landrace × Yorkshire, average body weight, 9.45 ± 0.4 kg) were divided into four groups with six replicates per group and one piglet per replicate according to similar weight and parity, with a ratio of males to females of 1:1. Each piglet was kept in an individual enclosure with 1.0 m^2^ of area per piglet. During the experiment, feed and water were freely provided, and disinfection and immunization were conducted in accordance with the routine procedures of the pig farm. The trial period was 28 days, involving a 7-day pretrial period and a 21-day formal period.

A basal diet of “corn–wheat–soybean” was used according to the nutritional requirements for weaned piglets recommended by the NRC (2012). The nutritional formula of the basal diet is shown in Table 1. The experimental groups and diets were as follows: (1) CON, fed with a normal diet; (2) RES, fed with a normal diet + 300 mg/kg RES; (3) DON, fed with a wheat diet containing 2.65 mg/kg DON/deoxynivalenol; and (4) DON + RES, fed with a wheat diet containing DON + 300 mg/kg RES.

### 2.2. Preparation of DON-Contaminated Wheat and Measurement of Mycotoxin Content

DON-contaminated wheat and dose selection were prepared as previously described [19,20]. The contents of DON in wheat and feed were determined by immunoaffinity column purification-high performance liquid chromatography according to a previous study [21]. The contents of aflatoxin B1, zearalenone, and T-2 toxin were determined through liquid chromatography–tandem mass spectrometry [22]. The mycotoxins in mildewed wheat were as follows: DON, 8260.14 μg/kg; aflatoxins B_1_, 2.78 μg/kg; zearalenone, 375.56 μg/kg; and T-2 toxin, 212.64 μg/kg. The content of mycotoxin in feed and the hygienic standard of feed (GB13078-2017) are shown in Table 2.

### 2.3. Experimental Design and Sample Collection

Each piglet was weighed at the beginning and end of the feeding and slaughtering trials, and feed consumption was recorded daily during the trial period. The average daily gain, average daily feed intake, and the feed-to-weight ratio of each piglet were calculated at the end of the experiment. At the end of the experiment, all the pigs were slaughtered and dissected. The heart, liver, spleen, and kidney were cleaned with 4 °C saline solution, and surface moisture was drained for weighing. The organ index was calculated as organ weight (g)/live weight (kg).

### 2.4. Morphological Analysis of the Intestinal Tract

The morphological analysis was conducted according to Wang et al. A segment of proximal jejunum approximately 1 cm was selected and rinsed with saline solution [23]. After the surface liquid was drained, the sample was fixed in 10% formalin at 4 °C. After dehydration with a concentration gradient of ethanol, the samples were made transparent via xylene treatment and embedded in paraffin, then sliced using an RM2135 rotary microtome (Leica, Wetzlar, Germany) followed by hematoxylin–eosin staining (HE) and neutral gum sealing. Quantitative analyses of villi and crypts were performed using a Qwin image analyzer (Leica). The vertical distance from the top of the villi to the crypt opening was regarded as the height of villi, while the vertical distance from the crypt opening to the crypt base was regarded as the depth of the crypt. Three HE-stained sections were selected from each sample. Three fields were randomly selected for each section. At least 20 measurements were taken for each crypt and villus measurement per pig. An average value was calculated.

### 2.5. Measurement D-Lactic Acid and Diamine Oxidase (DAO) in the Plasma

The activity of DAO and the concentration of D-lactic acid were determined using kits from Nanjing Jiancheng Bioengineering Institute (Nanjing, China) according to the kit instructions.

### 2.6. Measurement of the Redox Status of Intestinal Mucosa

The activities of superoxide dismutase (SOD), glutathione peroxidase (GSH-Px), and the content of malondialdehyde (MDA) in the jejunal mucosa were determined using ELISA kits (Nanjing Jiancheng Bioengineering Institute, Nanjing, China) according to the previous studies [24,25].

### 2.7. Extraction of Mitochondria from Intestinal Mucosa

The extraction of mitochondria was conducted according to the previous study [26]. Approximately 0.5 g of jejunal mucosa tissue was homogenized in precooled MSH buffer (10 mmol/L HEPES containing 200 mmol/L mannitol, 70 mmol/L sucrose, 1.0 mmol/L EGTA, and 2.0 mg/mL serum albumin) and centrifuged at 1000× *g* at 4 °C for 10 min. The supernatant was then collected at 3500× *g* at 4 °C and left still for 10 min to obtain mitochondrial deposition. The protein concentration was determined using the BCA method [27].

### 2.8. ROS Levels in Mitochondria of the INTESTINAL Mucosa

The intestinal mucosal mitochondria were treated with 2 μmol/L 2′,7′-dichlorofluorescein diacetate, which could pass through the outer mitochondrial membrane. They were incubated at room temperature for 20 min, and then the fluorescence intensity was determined using a fluorescence microplate reader [28].

### 2.9. Measurement of the Intestinal Mitochondrial Membrane Potential (ΔΨm)

The ΔΨm was determined using JC-1 ΔΨm detection kits (Beyotime Institute of Biotechnology, Haimen, China) according to the manufacturer’s instructions [29]. The isolated mitochondria were suspended in 0.5 mL medium containing 5 mmol/L JC-1. After mixing, the fluorescence was immediately measured using an FLx800 fluorescence microplate reader (BioTek, Winooski, VT, USA). When the mitochondrial membrane potential was high, JC-1 aggregates in the matrix of the mitochondria formed a polymer, which produced a red fluorescence; when the mitochondrial membrane potential was low, JC-1 could not aggregate in the matrix of mitochondria, forming a monomer, which produced a green fluorescence. The ΔΨm of intestinal mitochondria could therefore be determined by the fluorescence ratio of aggregates to monomers.

### 2.10. Data Processing and Analysis

The data were processed and analyzed as a 2 × 2 factorial arrangement by ANOVA using SPSS statistical software, version 26.0 (SPSS, Chicago, IL, USA). The statistical model includes the effects of DON (challenged or not challenged), resveratrol (supplemented or not supplemented), and their interaction. Data are shown as means ± SD. The differences between means were analyzed using Duncan’s multiple-range tests. Significance was considered as *p* < 0.05.

## 3. Results

### 3.1. The Effect of Dietary RES on the Growth Performance of Piglets after DON Challenge

As shown in Table 3., compared with the CON group, the final body weight, average daily gain, and average daily feed intake were significantly decreased (*p* < 0.05) by DON. Meanwhile, the dietary addition of RES in the DON group significantly increased average daily feed intake (*p* < 0.05), and there was an increasing trend (0.05 < *p* < 0.10) in the final body weight in the DON + RES group relative to the DON group. There was no significant resveratrol−DON interaction on the growth performance of piglets.

### 3.2. The Effect of Dietary RES on the Organ Index of Piglets after DON Challenge

Table 4 shows that the organ indices of the liver and kidney of piglets in the DON group were significantly greater than those in the CON group (*p* < 0.05), indicating that DON treatment caused enlargements of the liver and kidney. Meanwhile, no significant difference was found between the DON group and the RES group on the organ index of piglets. There was no significant resveratrol−DON interaction on the organ index of piglets.

### 3.3. The Effect of Dietary RES on the Jejunal Morphology of Piglets after DON Challenge

As shown in Table 5., in comparison with the CON piglets, the DON-contaminated diet significantly (*p* < 0.001) decreased the height of the jejunal villus and the ratio of villus height/crypt depth, and increased (*p* = 0.034) the crypt depth. Meanwhile, dietary supplementation with RES in the DON group exerted a protective effect on jejunal morphology by significantly increasing (*p* = 0.005, *p* = 0.006) the villus height and the ratio of villus height/crypt depth in the jejunum of piglets. There was a significant resveratrol−DON interaction (*p* = 0.009) on the villus height/crypt depth of piglets.

### 3.4. The Effects of RES on the Jejunal Permeability of Piglets after DON Challenge

Table 6 shows that the levels of DAO and D-lactic acid in the plasma were significantly elevated (*p* < 0.05) in the DON group when compared with the CON group. Meanwhile, no significant difference (*p* > 0.05) was found between the DON group and the DON + RES group on the levels of DAO and D-lactic acid. There was no significant resveratrol−DON interaction on the jejunal permeability of piglets.

### 3.5. The Effects of RES on Antioxidant Activities in the Jejunum of Piglets after DON Challenge

When compared with the control group, DON significantly decreased the activities of SOD and T-AOC in the jejunal mucosa of piglets (Table 7; *p* < 0.05), while the content of malondialdehyde (MDA) was raised remarkably (*p* < 0.05) in the DON group. Nevertheless, the addition of RES could significantly alleviate the negative effect of DON on jejunal SOD, T-AOC, and MDA (*p* < 0.05). There was no difference in GSH-Px activity among the groups (*p* > 0.05). Regarding the T-AOC activity and MDA content, there were interactions between RES and DON treatments in the jejunum of piglets (*p* < 0.05).

### 3.6. The Effects of RES on Mitochondrial ROS Levels and Membrane Potential in the Jejunum of Piglets after DON Challenge

As shown in Table 8, the ROS level of the jejunal mitochondria was evidently induced in the DON group (*p* < 0.05), while the mitochondrial membrane potential was significantly reduced (*p* < 0.05). Meanwhile, the addition of RES in the DON group inhibited the level of ROS (*p* < 0.05) and enhanced the membrane potential (*p* < 0.05). However, the ROS level between the DON + RES and CON groups still exhibited a significant difference (*p* < 0.05), suggesting that the addition of RES in the DON group could not completely eliminate the adverse effects of DON. Moreover, there was an interaction between RES addition and DON treatment on the ROS level of piglets (*p* < 0.05).

## 4. Discussion

A DON-contaminated diet usually affects growth performance by reducing the feed intake of piglets [30,31]. RES is a natural polyphenol belonging to the phytoalexin family. It has been shown to possess the ability to improve the antioxidant capacity, immune function, intestinal morphology, growth and reproductive performances, and meat quality of pigs [32,33,34]. In this study, the average daily gain and average daily feed intake were significantly decreased by the addition of DON, while supplementation with RES in the DON-contaminated group significantly increased the average daily feed intake, suggesting that RES alleviated the adverse effects of DON on weaned piglets. However, the increase in the average daily gain and the reduction of feed/gain by RES were not significant, which might be related to the experimental period of the study. Furthermore, in contrast with a previous study [32], the addition of RES to the basic diet had no significant effect on growth performance, which might be related to feed composition, as well as the age or weaning weight of piglets [35].

The organ index is essential for judging the functional status of organs. DON may cause damage to the liver, spleen, thymus, kidney, and intestinal epithelial cells [36,37]. Supplementation with RES in the diet significantly improved the bursa and spleen indices of black bone chickens under heat stress [38], and protected the liver from injury under oxidative stress in mice [39,40]. Similarly, our results showed that DON caused no significant damage to the heart and spleen, but led to the swelling of the liver and kidney. Meanwhile, the addition of RES to the DON group had no significant effect on the organ index, which could result from the different animal models used in experiments.

The integrity of the structure and morphology of the intestinal mucosa is the basis for normal digestion and the absorption of nutrients. DON exposure destroyed the integrity of the intestinal barrier, as well as the protein structural organization of intestinal epithelial cells, leading to oxidative stress, inflammation, and apoptosis. The degree of injury from DON treatment to the structure and morphology of intestinal villus epithelial cells in pigs was influenced by the dose and feeding period [41,42]. It has been shown that RES increased the height of the jejunum villi, decreased the crypt depth, and decreased the number of apoptotic cells, improving the intestinal function of piglets [43,44]. We verified that DON significantly decreased the height of the jejunal villi and the ratio of the jejunal villi height/crypt depth, which is consistent with a previous study [45]. Moreover, the dietary addition of RES in the DON group could ameliorate DON-induced intestinal morphology disorder by increasing the villus height and the ratio of the villus height/crypt depth of piglets when compared with the DON group, indicating that supplementing with RES in the DON-contaminated diet could remarkably alleviate the adverse effects of DON and improve the index of intestinal morphology. Unlike previous results [44], the addition of RES in the basal diet had no positive effect on jejunum morphology, which might result from the diverse feed composition and the day age of the piglets. It is therefore possible that RES may only play key roles under stress conditions in piglets.

External stress can damage the integrity of the intestinal epithelial barrier, resulting in the metabolic dysfunction of nutrients. The levels of DAO and D-lactic acid in plasma usually reflect the degree of damage to the intestinal barrier, also called intestinal permeability. DON could cause damage to the intestinal mucosa, changing the permeability of the intestinal mucosa. The activities of DAO and the content of D-lactic acid in piglets are increased after DON challenge [46]. Cao et al. also demonstrated that RES supplementation in the diet of piglets reversed the decline of occludin, claudin-1, and zonula occludens-1 protein levels in the jejunum induced by diquat, thus affecting intestinal mucosal permeability and improving intestinal barrier function [29]. Our data showed that the levels of DAO and D-lactic acid in plasma were significantly increased in a diet containing DON, which is consistent with the previous results [47]. However, the activities of DAO and the concentration of D-lactic acid in the plasma of DON-contaminated diets plus RES were not significantly different from those in the DON group. The possible reasons for the different results with Cao et al. could result from the differences in the initial body weight of the piglets and the intestinal injury model. Therefore, further research, such as the assessment of genes and the protein expression of the tight junction, would help reveal the underlying mechanism of RES on the intestinal barrier of piglets.

DON could disrupt the normal function of mitochondria and cause the release of free radicals, which induce lipid peroxidation and affect the integrity of cell membranes and the signaling of the redox cycle, resulting in increases in ROS, MDA, and TBARS, as well as decreases in the activities of antioxidant enzymes, such as glutathione and SOD [9,48]. DON can also induce mitochondrial injury by reducing mitochondrial membrane potential and induce apoptosis by up-regulating the expressions of apoptosis-related factors, such as caspase-3, caspase-8, and caspase-9 [9,49,50]. It was reported that diets supplemented with RES in piglets alleviated the oxidative stress induced by diquat through an increase in T-AOC and a decrease in H_2_O_2_ and MDA in jejunum mucosa [29]. It was also shown that RES significantly increased the activity of glutathione and SOD in the liver, and decreased the level of MDA in the serum of weaned piglets [44]. In the present study, the levels of SOD and T-AOC in the jejunum mucosa of piglets was significantly declined due to DON in the feed, while the level of MDA significantly increased. Furthermore, the ROS level in mitochondria in the DON group was significantly increased, while the mitochondrial membrane potential in jejunum was decreased when compared with the control group. Compared with DON group, RES increased the activities of SOD and T-AOC, and the mitochondrial membrane potential, and decreased the levels of ROS and MDA of jejunum. Nevertheless, although RES significantly reduced the level of mitochondrial ROS production in the DON group, it still maintained a relatively high level of ROS when compared with the CON group, suggesting that the addition of RES merely increased the antioxidant ability of weaned piglets to a certain degree. Therefore, if the DON content in the diet was too high, the mitochondrial function would still be damaged.

## 5. Conclusions

In conclusion, the present study demonstrated that supplemental resveratrol attenuated oxidative stress, and improved mitochondrial function and intestinal morphology in DON-challenged piglets. This study showed that resveratrol might serve as an effective additive to treat intestinal disorders involved in DON-induced growth-retardation in piglets.

## Figures and Tables

**Table 1:** Composition and nutrient levels of the basal diet.

Ingredients	g/kg	Composition (Analyzed Except for Digestible Energy) ^b^	g/kg
Maize	363	Digestible energy, MJ/Kg	14.2
Wheat	300	Crude protein	208
Soybean meal	250	Calcium	7.6
Fish meal	30	Total phosphorus	6.6
Soybean oil	15	Available phosphorus	4.6
Limestone meal	05	Sodium	2.5
Dicalcium phosphate	11	Methionine and Cystine	4.5
Sodium chloride	03	Lysine	12.8
L-Lysine HCl	02		
DL-Methionine	0.1		
Premix ^a^	2.0		

^a^ Per kilogram of diet provided: vitamin A 8 750 IU, vitamin D_3_, 2500 IU, vitamin E 25 IU, vitamin K_3_ 2.5 mg, vitamin B_1_ 2.5 mg, vitamin B_2_ 6.25 mg, vitamin B_6_ 2.5 mg, vitamin B_12_ 25 μg, d-biotin 100 μg, folic acid 1.25 mg, nicotinamide 25 mg, d-pantothenic acid 12.5 mg, Zn 80 mg, Fe 100 mg, Cu 20 mg, Mn 20 mg, I 0.14 mg, Se 0.3 mg, xylanase 200 FXU. ^b^ Digestible energy was calculated from data provided by the Feed Database in China (2012).

**Table 2:** Mycotoxin content in feed (μg/kg).

Toxin	Negative Control Diet	DON ^1^	Hygienic Standard for Feed ^2^
DON	150.16	2650.25	≤1000
Aflatoxins B_1_	1.71	2.73	≤10
Zearalenone	30.25	141.84	≤150
T-2 toxin	50.18	118.76	≤500

^1^ DON contaminated diet. ^2^ General Administration of Quality Supervision, Inspection and Quarantine tPsRoC (GAQSIQ) GB/T 13078-2017, Hygienical Standard for Feeds. Standards Press of China; Beijing, China: 2017.

**Table 3:** The effects of RES (RES) on the growth performance of piglets.

Items	Group Name				*p*-Value		
	CON ^1^	RES ^2^	DON	DON + RES	RES	DON	Interaction
Initial body weight, kg	9.45 ± 0.24	9.44 ± 0.22	9.43 ± 0.25	9.44 ± 0.21	0.992	0.896	0.866
Final body Weight, kg	20.48 ± 0.69 ^a^	20.84 ± 0.67 ^a^	19.16 ± 0.70 ^b^	19.81 ± 0.65 ^ab^	0.082	<0.001	0.602
Average daily gain, g	525.11 ± 32.44 ^ab^	543.02 ± 40.35 ^a^	463.35 ± 34.77 ^c^	493.69 ± 29.43 ^bc^	0.102	0.001	0.663
Average daily feed intake, g	745.06 ± 35.56 ^a^	747.22 ± 25.21 ^a^	644.55 ± 22.94 ^c^	699.66 ± 24.98 ^b^	0.114	<0.001	0.160
Ratio (feed/gain)	1.42 ± 0.10	1.38 ± 0.12	1.44 ± 0.12	1.42 ± 0.12	0.562	0.538	0.819

^1^ control group (CON), piglets fed with a basal diet; ^2^ RES group (RES), piglets fed with a basal diet + 300 mg/kg RES; DON group (DON), piglets fed with a basal diet containing 2.65 mg/kg DON; DON + RES group (DON + RES), piglets fed with a basal diet containing 2.65 mg/kg + 300 mg/kg RES. ^a,b,c^ Different letters in the same row indicate significant differences (*p* < 0.05), no letters in the same line or the same letters in the data shoulder indicate that the difference is not significant (*p* > 0.05) (the same as in the table below).

**Table 4:** The effects of RES on the organ index of piglets.

Items	Group Name				*p*-Value		
	CON	RES	DON	DON + RES	RES	DON	Interaction
Heart, g/kg	3.81 ± 0.22	3.84 ± 0.25	3.91 ± 0.25	3.83 ± 0.17	0.814	0.665	0.552
Liver, g/kg	20.25 ± 1.62 ^b^	20.29 ± 1.52 ^b^	23.24 ± 1.54 ^a^	22.72 ± 1.36 ^a^	0.699	<0.001	0.660
Spleens, g/kg	1.79 ± 0.12	1.79 ± 0.13	1.91 ± 0.18	1.89 ± 0.15	0.837	0.089	0.859
Kidney, g/kg	3.53 ± 0.20 ^b^	3.51 ± 0.19 ^b^	4.03 ± 0.27 ^a^	3.83 ± 0.22 ^a^	0.239	<0.001	0.331

^a,b^ Different letters in the same row indicate significant differences (*p* < 0.05), no letters in the same line or the same letters in the data shoulder indicate that the difference is not significant (*p* > 0.05).

**Table 5:** The effects of RES on the jejunal morphology of piglets.

Items	Group Name				*p*-Value		
	CON	RES	DON	DON + RES	RES	DON	Interaction
Villus height, μm	501.13 ± 28.43 ^a^	513.40 ± 21.11 ^a^	426.06 ± 31.98 ^b^	482.92 ± 24.38 ^a^	0.005	<0.001	0.055
Crypt depth, μm	180.01 ± 12.62 ^b^	183.58 ± 12.39 ^ab^	198.98 ± 13.17 ^a^	187.96 ± 12.01 ^ab^	0.475	0.034	0.170
Villus height/crypt depth	2.79 ± 0.20 ^a^	2.80 ± 0.14 ^a^	2.14 ± 0.12 ^b^	2.58 ± 0.23 ^a^	0.006	<0.001	0.009

^a,b^ Different letters in the same row indicate significant differences (*p* < 0.05), no letters in the same line or the same letters in the data shoulder indicate that the difference is not significant (*p* > 0.05).

**Table 6:** The effects of RES on the jejunal permeability of piglets.

Items	Group Name				*p*-Value		
	CON	RES	DON	DON + RES	RES	DON	Interaction
Plasma D-lactic acid, μmol/L	6.05 ± 0.69 ^b^	5.90 ± 0.73 ^b^	7.25 ± 0.78 ^a^	6.43 ± 0.55 ^ab^	0.100	0.006	0.250
Plasma DAO, U/L	5.29 ± 0.84 ^b^	5.35 ± 0.66 ^b^	6.62 ± 0.72 ^a^	6.01 ± 0.42 ^ab^	0.320	0.002	0.240

^a,b^ Different letters in the same row indicate significant differences (*p* < 0.05), no letters in the same line or the same letters in the data shoulder indicate that the difference is not significant (*p* > 0.05).

**Table 7:** The effects of RES on antioxidant enzymes activities and MDA content in the jejunum of piglets.

Items	Group Name				*p*-Value		
	CON	RES	DON	DON + RES	RES	DON	Interaction
SOD, U·mg^−1^ protein	100.17 ± 8.98 ^a^	104.33 ± 8.73 ^a^	82.17 ± 7.14 ^b^	96.33 ± 7.31 ^a^	0.012	0.001	0.145
GSH-Px, U·mg^−1^ protein	88.17 ± 8.06	91.17 ± 7.14	82.83 ± 6.74	87.00 ± 6.81	0.237	0.122	0.845
T-AOC, U·mg^−1^ protein	2.07 ± 0.21 ^a^	2.14 ± 0.28 ^a^	1.40 ± 0.23 ^b^	1.88 ± 0.19 ^a^	0.008	<0.001	0.041
MDA, nmol·g-1 protein	0.94 ± 0.19 ^b^	0.89 ± 0.13 ^b^	1.57 ± 0.16 ^a^	1.01 ± 0.12 ^b^	<0.001	<0.001	<0.001

^a,b^ Different letters in the same row indicate significant differences (*p* < 0.05), no letters in the same line or the same letters in the data shoulder indicate that the difference is not significant (*p* > 0.05).

**Table 8:** The effect of RES on mitochondrial ROS production and mitochondrial membrane potential in the piglet jejunum.

Items	Group Name				*p*-Value		
	CON	RES	DON	DON + RES	RES	DON	Interaction
ROS level (Fold change)	1.00 ± 0.38 ^c^	0.90 ± 0.19 ^c^	5.73 ± 0.82 ^a^	2.63 ± 0.39 ^b^	<0.001	<0.001	<0.001
Membrane potential ΔΨm (Fold change)	1.00 ± 0.19 ^a^	1.04 ± 0.18 ^a^	0.42 ± 0.12 ^c^	0.76 ± 0.16 ^b^	0.011	<0.001	0.032

^a,b,c^ Different letters in the same row indicate significant differences (*p* < 0.05), no letters in the same line or the same letters in the data shoulder indicate that the difference is not significant (*p* > 0.05).

## Data Availability

The authors confirm that the data supporting the findings of this article are available.
